# Plant strategies for greatest height: tapering or hollowing

**DOI:** 10.1038/s41598-023-45468-7

**Published:** 2023-10-24

**Authors:** Tohya Kanahama, Motohiro Sato

**Affiliations:** 1 Graduate School of Engineering, Hokkaido University, Kita 13, Nishi 8, Kita-Ku, Sapporo, 060-8628 Japan; 2 Faculty of Engineering, Hokkaido University, Kita 13, Nishi 8, Kita-Ku, Sapporo, 060-8628 Japan

**Keywords:** Plant sciences, Engineering

## Abstract

The tapered form and hollow cross-section of the stem and trunk of wild plants are rational mechanical approaches because they facilitate the plant simultaneously growing taller for photosynthesis and supporting its own weight. The purpose of this study is to clarify the advantages and disadvantages of tapering and hollowing from the perspective of the greatest probable height before self-buckling. We modelled woody plants using tapering or hollow cantilevers, formulated the greatest height before self-buckling, and derived a theoretical formula for the greatest probable height considering tapering and hollowing. This formula theoretically explains why almost all plants exhibit a tapered form: it allows for a greater height at an earlier growth stage than a hollow cross-section.

## Introduction

All plants are equally affected by gravity; therefore, they must efficiently resist gravity to grow sufficiently tall and large for photosynthesis while adapting themselves to the environment in which they grow. Consequently, plant shapes are remarkably diverse. However, various plants have consistent scaling laws, possibly because they share a common advantage: “to efficiently resist gravity.” For example, Greenhill theoretically derived that the greatest height to which a tree can grow without self-weight buckling is proportional to its radius to the power of 2/3^1^. The validity of this theory was verified by McMahon, who confirmed that it accurately fits trees of various shapes^2,3^. Greenhill's scaling law, with its simplicity and applicability, has been used extensively in forest science and ecology^4–9^.

Other factors governing the greatest possible height, include hydraulic conditions^10,11^, wind effects^12,13^, and genetic factors^14^. However, Greenhill's height–diameter scaling law fits actual trees, which indicates that the greatest height derived based on the assumption of self-weight buckling includes a scaling law that is broadly applicable to trees.

In addition, a wide observation of tree forms reveals a common geometrical feature: a tapered form that becomes sharper from the base to the tip^7,15–23^. This tapered form reduces weight at the free end and improves the bending rigidity near the base; it is a structure that efficiently increases resistance to gravity. However, in many studies, the theoretical scaling law that links the tapered form and greatest height for self-buckling has not been clarified because the calculation model was limited, and the finite element method was used as an analytical method^7,15,16^. Previously, we extended the calculation model from a cylinder to a truncated cone, which was arbitrarily tapered to deeply consider the effect of this form, and derived the greatest height for self-weight buckling^17^. In the study, we formulate a new scaling law for the degree of taper and determine the greatest height, in addition to a scaling law for the radius, which is the same as Greenhill's law. Furthermore, the greatest height of a tapered cylinder is found to be approximately 1.5 times that of a cylinder without tapering.

As described above, trees that are particularly tall and large can grow to exceed human height by skillfully distributing their weight and increasing their resistance to gravity through the tapered form of their trunks, despite their dense cross-sections, heavy branches, and leaves. In addition to the tapered form, one effective strategy for reducing weight and securing bending rigidity is to contain a hollow cross-section. For a given amount of material, increasing the radius of a solid cylinder to increase its bending rigidity results in an increase in weight. However, simply reducing the radius to reduce the weight results in a decrease in the bending rigidity. This problem can be solved by adopting a hollow cylindrical structure^24–26^. In fact, bamboos, which are smaller in diameter than trees, simultaneously possess both a tapered form and hollow structure; presumably, this allows them to grow as tall as trees.

Therefore, for plants to gain height, the “tapering” and “hollowing” approaches can be considered, which both reduce the weight of the cross-section and ensure bending rigidity. However, in woody plants, their cross-sections are almost densely packed, and they only have a tapered form. This suggests that for plants that need height for photosynthesis, the introduction of a tapered shape, rather than hollow cross-section, is a superior means of achieving both stability and height. From a mechanical point of view, this insight leads to the elucidation of the “efficient gravity-resistance mechanism” that plants have retained through evolution while changing their shapes, and the “scaling law of woody plants” that can be applied to the estimation of biomass. Furthermore, this contribution can be applied to design and structural calculations for civil engineering structures because the functionally superior tapered form is widely used in engineering^7,27,28^. Moreover, it is important and interesting for forest managers, researchers studying plant strategies, and many more to clarify the mechanical stability of woody plants.

This study aims to derive a brief greatest-height formula for columns with arbitrary tapers and hollow sections without self-weight buckling, determine the gravity resistance mechanism of plant morphology, and clarify the gain and loss of the taper form and hollow sections in terms of the greatest height. The governing equations for a cantilever with a hollow section and tapered form of an arbitrary size are obtained from the equilibrium of forces after buckling under self-weight. By applying boundary conditions to the general solution, we solve the eigenvalue problem and derive a scaling law under the deadweight buckling constraint that is widely applicable to woody plants. Using this equation, we not only clarify the gains and losses of tapered and hollow cross-sections in terms of the greatest height but also discuss the gains and losses of both forms as growth strategies and clarify the mechanical advantages of both approaches. Moreover, this study is expected to provide ecological knowledge on the essence of plant morphology, such as the selection of hollow cross-sections and tapered forms.

## Methods

### Modelling woody plants

**Figure 1:**
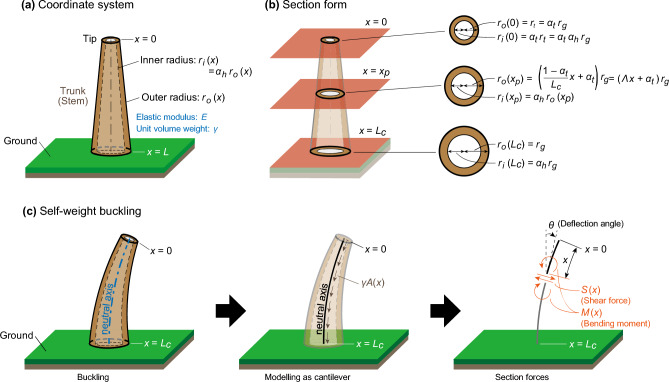
Modelling trees. (**a**) Coordinate system. It lies along the neutral axis, with $x=0$ at the free end and $x=L$ at the ground. ${r}_{i}(x)$ and ${r}_{o}(x)$ are the first-order functions of inner and outer radii with respect to $x$, respectively. (**b**) Section form. ${r}_{g}$ is the radius at ground $(x=L)$, and ${r}_{t}$ is that at tip $(x=t)$. To simplify the formulation, we introduced two non-dimensionless parameters, the taper ratio ${\alpha }_{t}={r}_{t}/{r}_{g}$ and hollow ratio ${\alpha }_{h}={r}_{i}(x)/{r}_{o}\left(x\right)$. (c) Buckling under self-weight. The woody plant is buckled by self-weight. The trees are modelled as cantilever, and by considering the equilibrium of forces, we can derive a governing equation.

The calculation model is a cantilever with a hollow section and tapered form of arbitrary size, as shown in Fig. 1a. The coordinate system lies along the neutral axis, with $x=0$ at the free end and $x=L$ at the fixed end. The parameters of the calculation model used in this study are listed in Table 1.

**Table 1:** Parameters of the calculation model.

a	Symbol	Unit	Remarks
Coordinate	$x$	[m]	$x=0$ at the tip and $x=L$ at the ground
Radius at tip ($x=0$)	${r}_{t}$	[m]	–
Radius at ground ($x=L$)	${r}_{g}$	[m]	–
Outer radius	${r}_{o}(x)$	[m]	${r}_{o}\left(x\right)=\left(\frac{1-{\alpha }_{t}}{{L}_{c}}x+{\alpha }_{t}\right){r}_{g}$
Inner radius	${r}_{i}(x)$	[m]	${r}_{i}\left(x\right)={\alpha }_{h}{r}_{o}\left(x\right)$
Taper ratio	${\alpha }_{t}$	–	${\alpha }_{t}={r}_{t}/{r}_{g}$
Hollow ratio	${\alpha }_{h}$	–	${\alpha }_{h}={r}_{i}(x)/{r}_{o}\left(x\right)$, constant
Simplification parameter	$\Lambda$	[1/m]	$\Lambda =\frac{1-{\alpha }_{t}}{{L}_{c}}$
Elastic modulus	$E$	[N/m^2^]	–
Unit volume weight	$\gamma$	[N/m^3^]	–
Height	$L$	[m]	–
Greatest height	${L}_{c}$	[m]	Length in buckling state

Figure 1b shows the section form at an arbitrary point ${x}_{p}$, tip ($x=0$), and fixed end ($x=L$). The calculation model in this study corresponds to a cone when the taper ratio is ${\alpha }_{t}=0$ and a cylinder when ${\alpha }_{t}=1$. Moreover, the section form is solid for the hollow ratio ${\alpha }_{h}=0$, and the volume of the cavity increases with the hollow ratio ${\alpha }_{h}$. When ${\alpha }_{h}=1$, the calculation model disappears. The real distribution of the hollow ratio in the vertical direction has been reported in various studies^29,30^. However, the hollow ratio ${\alpha }_{h}$ was assumed as constant in the vertical direction for simplification. In this study, the effects of branches and leaves were not considered because the focus was on the buckling characteristics of the trunk and stem under self-weight conditions, and the information about the ratio of total leaf mass to the above total mass in some bamboo species tends to be constant, as reported in previous studies^31^.

Moreover, the plants that have a hollow structure in stems and culms, such as bamboo, are known to have the ovalization and local buckling appearance caused by load perpendicular to the member axis, such as wind load, which are suppressed by discretely distributed nodes in the cross-section^24,25,32,33^. This effect of suppressing cross-sectional deformation has important role not only in the prevention of local buckling but also in making the structure behave as a beam structure that resists loading by bending deformation of the entire bamboo stem. In this study, considering that nodes have the effect of suppressing the cross-sectional deformation, based on the bamboo behaving as a "beam" that resists loading by bending deflection of the entire span, we focused on the self-weight buckling problem and derived its greatest height.

### Governing equation

The calculation model is a truncated cone cantilever, as shown in Fig. 1. When this model buckles (Fig. 1c), the shear force $S\left(x\right)$ can be obtained from the equilibrium of the forces at an arbitrary point $x$ as follows:

1 $$\begin{array}{c}S\left(x\right)=\gamma V\left(x\right)\mathrm{sin}\theta, \end{array}$$

where $V(x)$ is the volume from tip to position $x$ [m^3^] and $\theta$ is the deflection angle. $V(x)$ is given by

2 $$\begin{array}{c}V\left(x\right)=\left(1-{\alpha }_{h}^{2}\right)\left(\frac{1}{3}{\Lambda }^{2}{x}^{2}+\Lambda {\alpha }_{t}x+{\alpha }_{t}^{2}\right)\pi {r}_{g}^{2}x. \end{array}$$

The meanings and definitions of all the symbols, such as ${\alpha }_{h}$, ${\alpha }_{t}$, $\Lambda$ and ${r}_{g}$, is tabulated in Table 1. Because Eq. (2) is extremely complicated, the obtained governing differential equation cannot be solved if the formulation proceeds in this manner. Therefore, by introducing two calculation models illustrated in Fig. 5, the shear force $S(x)$ can be converted to a simpler form.

**Figure 2:**
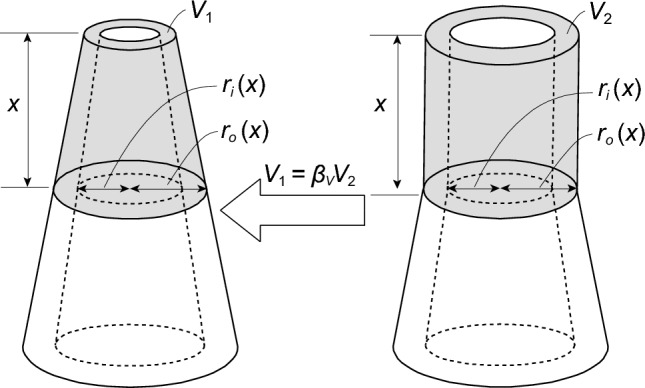
Variables required for volume correction coefficient ${\beta }_{V}$.

The volume of the shaded part ${V}_{1}$, which is shown on the left in Fig. 2, is given by Eq. (2). By contrast, the volume of the shaded part ${V}_{2}$ shown on the right in Fig. 2 is given by

3 $$\begin{array}{c}{V}_{2}\left(x\right)=\left(1-{\alpha }_{h}^{2}\right){\left(\Lambda x+{\alpha }_{t}\right)}^{2}\pi {r}_{g}^{2}x. \end{array}$$

We introduce a correction factor ${\beta }_{V}$, such that the volumes of the two models shown in Fig. 2 are equal. Multiplying the correction factor ${\beta }_{V}$ by ${V}_{2}$ easily determines the range of values for ${\beta }_{V}$. If ${V}_{1}={\beta }_{V}{V}_{2}$, the following equation is obtained:

4 $$\begin{array}{c}{\beta }_{V}\left(x\right)=\frac{1}{3}\left(1+\frac{{\alpha }_{t}{r}_{g}}{{r}_{o}\left(x\right)}+\frac{{{\alpha }_{t}}^{2}{r}_{g}^{2}}{{r}_{o}^{2}\left(x\right)}\right). \end{array}$$

Therefore, the volume correction factor ${\beta }_{V}$ in Eq. (4) is independent of the hollow ratio ${\alpha }_{h}$. Because it has the same formula as the volume correction factor in the truncated cone model, we can consider ${\beta }_{V}$ as a function that only varies with ${\alpha }_{t}$ and is independent of $x$^17^.

If the deflection angle is assumed to be extremely small, then the shear force $S(x)$ of Eq. (1) can be expressed as

5 $$\begin{array}{c}S\left(x\right)=\gamma \pi {\beta }_{V}\left(1-{\alpha }_{h}^{2}\right){\left(\Lambda x+{\alpha }_{t}\right)}^{2}{r}_{g}^{2}x\theta . \end{array}$$

The bending moment $M(x)$ at an arbitrary point $x$ can be obtained from the elastic curve equation as follows:

6 $$\begin{array}{c}M\left(x\right)= -EI(x)\frac{d\theta }{dx}, \end{array}$$

where $I$ is the moment of inertia [m^4^]. Using the relationship between the shear force and bending moment ($S=dM/dx$), the governing equation can be obtained from Eqs. (5) and (6) as follows:

7 $$\begin{array}{c}\frac{{d}^{2}\theta }{d{x}^{2}}+\frac{4\Lambda }{\left(\Lambda x+{\alpha }_{t}\right)}\frac{d\theta }{dx}+\frac{4\gamma {\beta }_{V}}{E\left(1-{\alpha }_{h}^{2}\right){\left(\Lambda x+{\alpha }_{t}\right)}^{2}{r}_{g}^{2}}x\theta = 0. \end{array}$$

Using the following equation, which is the same as that for the solid truncated cone model, we convert the governing equation (Eq. (7)) into the following form:

8 $$\begin{array}{c}\xi \left(x\right) = \left(\Lambda x+{\alpha }_{t}\right) \omega , \end{array}$$

where $\omega$ is a constant. Converting the variables in Eq. (7) using Eq. (8), the following converted governing equation is obtained:

9 $$\begin{array}{c}\frac{{d}^{2}\theta }{d{\xi }^{2}}+\frac{4}{\xi }\frac{d\theta }{d\xi }+\frac{4}{{\xi }^{2}}\left(\xi -\omega {\alpha }_{t}\right)\theta = 0. \end{array}$$

By defining the variable-converting parameter $\omega$ using the third term on the left-hand side, it can be rewritten as follows:

10 $$\begin{array}{c}\omega =\frac{1}{{\left(1-{\alpha }_{t}\right)}^{3}}\frac{1}{\left(1+{\alpha }_{h}^{2}\right)}\frac{\gamma {\beta }_{V}{L}_{c}^{3}}{E{r}_{g}^{2}}. \end{array}$$

## Calculation

### General solution of governing equation

The governing Eq. (9) is exactly the same as the formulation equation. For a solid truncated cone, its general solution is given by

11 $$\begin{array}{c}\theta \left(\xi \right) = \frac{1}{8}{\xi }^{-3/2}\left({J}_{\eta }\left(4\sqrt{\xi }\right)\Gamma \left(1+\eta \right) {c}_{1}+{J}_{-\eta }\left(4\sqrt{\xi }\right)\Gamma \left(1-\eta \right) {c}_{2}\right), \end{array}$$

where ${J}_{\eta }\left(x\right)$ is a Bessel function of the first kind of order *η*, ${c}_{1}$ and ${c}_{2}$ are arbitrary constants, and $\Gamma (x)$ is the Gamma function. Moreover, $\eta$ is given by

12 $$\begin{array}{c}\eta =\sqrt{16\omega {\alpha }_{t}+9}. \end{array}$$

By using the following boundary conditions for a cantilever, a conditional expression regarding $\xi$ can be introduced.

13 $$\begin{array}{c}\left\{\begin{array}{c}\frac{d\theta }{dx}=0 \left(at ~x=0\right) \\ \theta =0 \left(at~x={L}_{c}\right) \end{array}.\right. \end{array}$$

The governing equation in Eq. (10) and order $\eta$ of the Bessel function shown in Eq. (12) are exactly the same as those in the solid truncated cone model, except for the definition of $\omega$. Therefore, as in the formulation of a solid tapered column^17^, the following conditional expression for $\xi$ can be obtained using the boundary conditions on the fixed-end side:

14 $$\begin{array}{c}{J}_{\eta }\left(4\sqrt{\xi }\right)={J}_{-\eta }\left(4\sqrt{\xi }\right)=0. \end{array}$$

When order $\eta$ is an integer, $\xi$ that satisfies Eq. (14) can be uniquely defined. Based on Eq. (14) being derived from the boundary condition at the fixed end (Eq. (13)), we obtain the greatest height equation as follows:

15 $$\begin{array}{c}{L}_{c}={\left(\frac{{j}_{\eta ,1}^{2}}{32{\beta }_{V}}{\left(1-{\alpha }_{t}\right)}^{3}\right)}^{1/3}{\left(2\frac{E}{\gamma }{r}_{g}^{2}\left(1+{\alpha }_{h}^{2}\right)\right)}^{1/3}, \end{array}$$

where ${j}_{\eta ,1}$ is the first zero point in the Bessel function of the first kind. Moreover, the greatest height ${L}_{ch}$ of the non-tapering hollow cylinder is given by^26^

16 $$\begin{array}{c}{L}_{ch}={{\left(1+{\alpha }_{h}^{2}\right)}^{1/3}\left(2\frac{E}{\gamma }{r}_{g}^{2}\right)}^{1/3}. \end{array}$$

Greenhill^1^ derived the greatest height ${L}_{cs}$ of a solid cylinder as follows:

17 $$\begin{array}{c}{{L}_{cs}=\left(2\frac{E}{\gamma }{r}_{g}^{2}\right)}^{1/3}. \end{array}$$

Moreover, using the function $f({\alpha }_{h})$ regarding the hollow ratio ${\alpha }_{h}$, the coefficient part ${\left(1+{\alpha }_{h}^{2}\right)}^{1/3}$ of Eq. (16) can be rewritten as follows:

18 $$\begin{array}{c}f\left({\alpha }_{h}\right)={\left(1+{\alpha }_{h}^{2}\right)}^{1/3}. \end{array}$$

Using Eqs. (17) and (18), Eq. (15) can be rewritten as

19 $$\begin{array}{c}{L}_{c}={\left(\frac{{j}_{\eta ,1}^{2}}{32{\beta }_{V}}{\left(1-{\alpha }_{t}\right)}^{3}\right)}^{1/3}f\left({\alpha }_{h}\right){L}_{cs}={\left(\frac{{j}_{\eta ,1}^{2}}{32{\beta }_{V}}{\left(1-{\alpha }_{t}\right)}^{3}\right)}^{1/3}{L}_{ch}. \end{array}$$

For simplification, we converted Eq. (19) as follows:

20 $$\begin{array}{c}{L}_{c}={\left(\frac{{j}_{\eta ,1}^{2}}{32{\beta }_{V}}{\left(1-{\alpha }_{t}\right)}^{3}\right)}^{1/3}f\left({\alpha }_{h}\right){L}_{cs}=f\left({\alpha }_{t}\right)f\left({\alpha }_{h}\right){L}_{cs}, \end{array}$$

where $f\left({a}_{t}\right)$ is a function that considers the effect of taper ratio ${\alpha }_{t}$. From these equations, the greatest height of the hollow truncated cone model ${L}_{c}$ is expressed in the form of multiple coefficients for considering the effect of tapering and hollowing on the greatest height of a solid cylinder ${L}_{cs}$.

### Numerical method solving the greatest height equation

**Figure 3:**
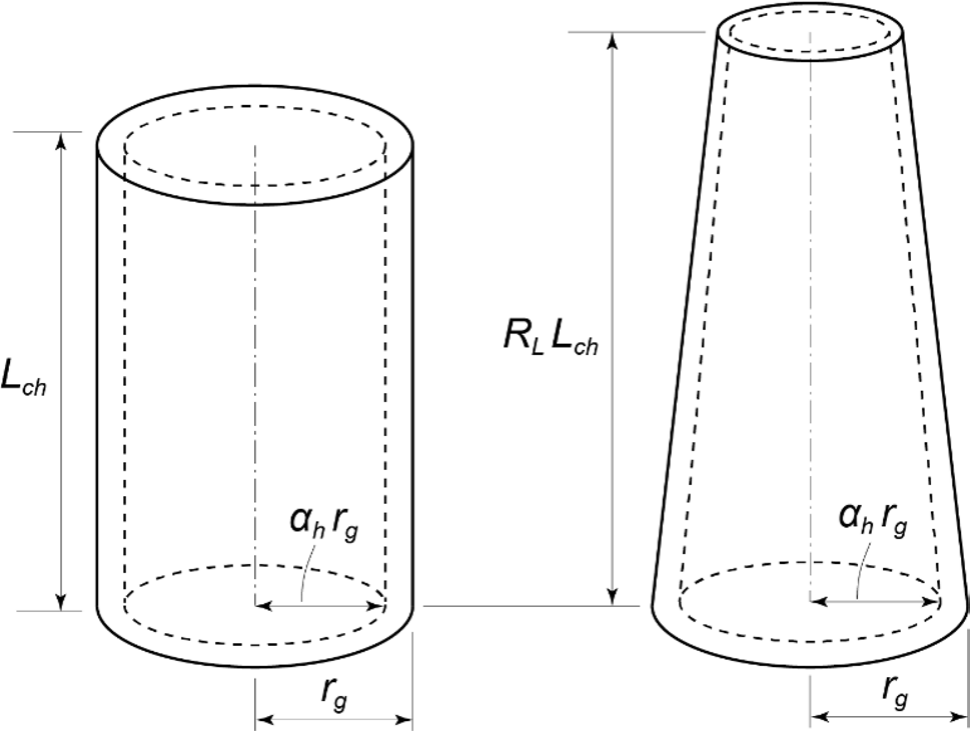
Variables required for critical height ratio of hollow model.

Equation (15) includes the greatest height ${L}_{c}$ of order $\eta$ of the Bessel function of the first kind, applied to integer conditions; handling this equation is very difficult. To solve this problem, using the same method as for the formulation of the solid truncated cone, the coefficient in Eq. (24) was derived considering the mechanical properties of the two calculation models (Fig. 3).

We considered two models with the same fixed-end radius ${r}_{g}$, hollow ratio ${\alpha }_{h}$, unit volume weight $\gamma$, and elastic modulus $E$, as shown in Fig. 3, differing only in the taper ratio ${\alpha }_{t}$. In this case, the greatest height of the tapered model is higher than that of the non-tapered model^17^. Based on this, we define the greatest height of the non-tapered model as ${L}_{ch}$ and that of the tapered model as ${L}_{c}$, and introduce the greatest height ratio ${R}_{L}$ of both as follows:

21 $$\begin{array}{c}{R}_{L}=\frac{{L}_{c}}{{L}_{ch}}. \end{array}$$

${R}_{L}$ is always ${R}_{L}\ge 1$ owing to the relationship of the greatest height. Moreover, by substituting Eq. (10) into Eq. (12), the following equation is obtained:

22 $$\begin{array}{c}16{\alpha }_{t}\frac{1}{{\left(1-{\alpha }_{t}\right)}^{3}}\frac{1}{\left(1+{\alpha }_{h}^{2}\right)}\frac{\gamma {\beta }_{V}{L}_{c}^{3}}{E{r}_{g}^{2}}+9={\eta }^{2}. \end{array}$$

By substituting Eqs. (20) and (21) into Eq. (22), the following equation is obtained:

23 $$\begin{array}{c}{R}_{L}\left({\alpha }_{t}\right)=\left(1-{\alpha }_{t}\right){\left\{\frac{{\eta }^{2}-9}{32{\alpha }_{t}{\beta }_{V}}\right\}}^{1/3}, \end{array}$$

which is independent of the hollow ratio ${\alpha }_{h}$ and the same as the formulation for a solid truncated cone. Therefore, using the result of the previous formulation^17^, Eq. (23) can be rewritten as

24 $$\begin{array}{c}{R}_{L}\left({\alpha }_{t}\right)=f\left({\alpha }_{t}\right)\approx \frac{1}{2}{\alpha }_{t}^{2}-{\alpha }_{t}+\frac{3}{2}. \end{array}$$

Using the above equation, we obtain a function of the taper ratio ${\alpha }_{t}$. The greatest height of the hollow cylinder is expressed by multiplying the function of the hollow ratio ${\alpha }_{h}$ in Eq. (18) by that of the non-tapered hollow cylinder (Eq. (16)); that of the hollow truncated cone ${L}_{c}$ can be calculated as follows:

25 $$\begin{array}{c}{L}_{c}=f\left({\alpha }_{t}\right)f\left({\alpha }_{h}\right){\left(2\frac{E}{\gamma }{r}_{g}^{2}\right)}^{1/3}=f\left({\alpha }_{t}\right)f\left({\alpha }_{h}\right){L}_{cs}, \end{array}$$

where $f({\alpha }_{t})$ and $f\left({\alpha }_{h}\right)$ are given by Eqs. (24) and (18), respectively. Equation (25) indicates that the greatest height for a hollow truncated cone can be obtained by multiplying the functions of ${\alpha }_{t}$ and ${\alpha }_{h}$ by the greatest height of the cylinder. Therefore, the height–diameter scaling law already verified by McMahon ^2,3^ is theoretically valid when considering tapered geometry and a hollow cross-section, as well as when the material is modeled as a cylinder. From the perspective of material properties, Eq. (25) implies that the density must be low and the material must be hard to increase the greatest height. Furthermore, from a geometrical perspective, rigidity must be maintained near the base, where the moment is large and high, by increasing the fixed-end radius while reducing the volume by tapering or making the cross-section hollow.

## Results

### Effect of tapering and hollowing on self-buckling characteristics in constant base radius

To survive, plants must compete with other plants for as much light as possible. We can observe this from “apical dominance" in which growth near the tip is prioritized, and the first stage of bamboo growth is culm height growth^34^. However, whereas weight reduction is effective in increasing the greatest height, density is closely related to the modulus of elasticity, and excessive weight reduction in the trunk may lead to reduced resistance to lateral loads, including wind, thereby increasing the risk of overturning^12,13,35^.

**Figure 4:**
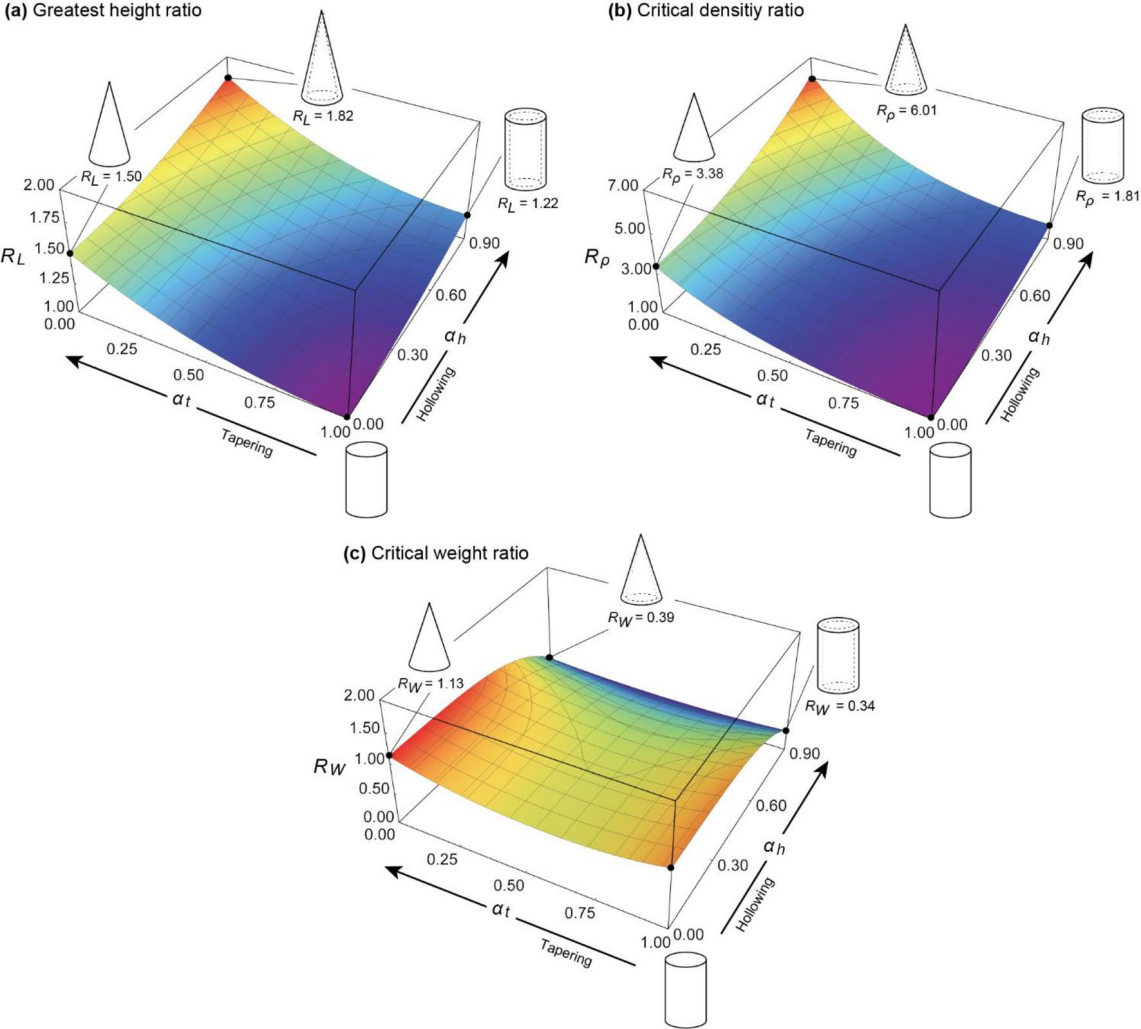
Effect of tapering and hollowing on self-buckling characteristics under constant radius conditions. (**a**) Greatest height ratio ${R}_{L}$. It is the ratio of the greatest height in tapering and hollowing models to those of non-tapering and hollowing models. (**b**) Critical density ratio ${R}_{\rho }$. It is the ratio of critical density in tapering and hollowing models to those of non-tapering and hollowing models. (**c**) Critical weight ratio ${R}_{W}$. It is the ratio of critical total weight in tapering and hollowing models to those of non-tapering and hollowing models.

Based on these factors, the effects of the taper ratio ${\alpha }_{t}$ and hollow ratio ${\alpha }_{h}$ on the self-weight buckling characteristics (greatest height and critical density) were first examined under the condition that the fixed-end radius ${r}_{g}$ is constant, as shown in Fig. 4 (a). The vertical axis shows the ratio of the self-weight buckling characteristic of a hollow truncated cone to that of a solid cylinder. The greatest height ratio ${R}_{L}({\alpha }_{t},{\alpha }_{h})$ and critical density ratio ${R}_{\rho }({\alpha }_{t},{\alpha }_{h})$ in the figure are given by the following equations, respectively:

26 $$\begin{array}{c}{R}_{L}=f\left({\alpha }_{t}\right)f\left({\alpha }_{h}\right), \end{array}$$

27 $$\begin{array}{c}R\rho =f{\left({\alpha }_{t}\right)}^{3}f{\left({\alpha }_{h}\right)}^{3}={R}_{L}^{3}. \end{array}$$

Moreover, the ranges of the taper and hollow ratios in the following numerical calculation examples were assumed to be ${0\le \alpha }_{t}\le 1$ and $0\le {\alpha }_{h}\le 0.9$, respectively, based on measurements of bamboo and trees^18–21,29,36–41^. The elastic modulus $E$ was constant, as shown in Fig. 4.

From this figure, when the fixed-end radius ${r}_{g}$ is kept constant, the greatest height can be increased up to approximately 1.2 times when only a hollow cross-section is employed and up to approximately 1.5 times when only a tapered form is employed. Furthermore, when both a tapered form and hollow cross-section are simultaneously employed, the greatest height can be increased to approximately 1.8 times that of a solid cylinder, thus confirming that both contribute to the greatest height increase. The tapered form is more effective than the hollow cross-section in terms of the greatest height that can be achieved when the fixed-end radius is kept constant.

The critical density (as shown in Fig. 4b) can be increased by a factor of up to 1.8 when only hollowing is employed and by up to 3.4 when only tapering is employed. Furthermore, the critical density can be increased up to approximately 6.0 times that of a solid cylinder when both a tapered shape and hollow cross-section are simultaneously employed, as is the case with bamboos. For the greatest height, the adoption of a tapered form is more effective than hollowing in increasing the greatest height in terms of the critical density.

Figure 4 (c) shows the ratio of the maximum allowable weight without self-buckling of the solid cylinder to that of the truncated cone. The critical total weight ratio ${R}_{W}({\alpha }_{t},{\alpha }_{h})$ on the vertical axis is given by

28 $$\begin{array}{c}{R}_{W}=f{\left({\alpha }_{t}\right)}^{3}f{\left({\alpha }_{h}\right)}^{3}\left(\frac{1}{3}\left({\alpha }_{t}^{2}+{\alpha }_{t}+1\right)\left(1-{\alpha }_{h}^{2}\right)\right). \end{array}$$

From Fig. 4c, when adopting the tapered form, the maximum allowable weight does not change significantly. However, when the best self-buckling characteristics were obtained (taper ratio ${\alpha }_{t}=0$), the maximum allowable weight was greater than that of the cylindrical column (${R}_{W}=1$). By contrast, in the case of adopting a hollow cross-section, the greatest allowable weight decreased with the hollowing of the cross-section by approximately 70% when ${\alpha }_{h}=0.9$.

### Effect of tapering and hollowing on self-buckling characteristics in constant volume

We can assume that the usable amount of biomass is limited and depends on the growth process. In this section, we consider the effects of tapering and hollowing on the self-buckling characteristics under constant volume conditions.

**Figure 5:**
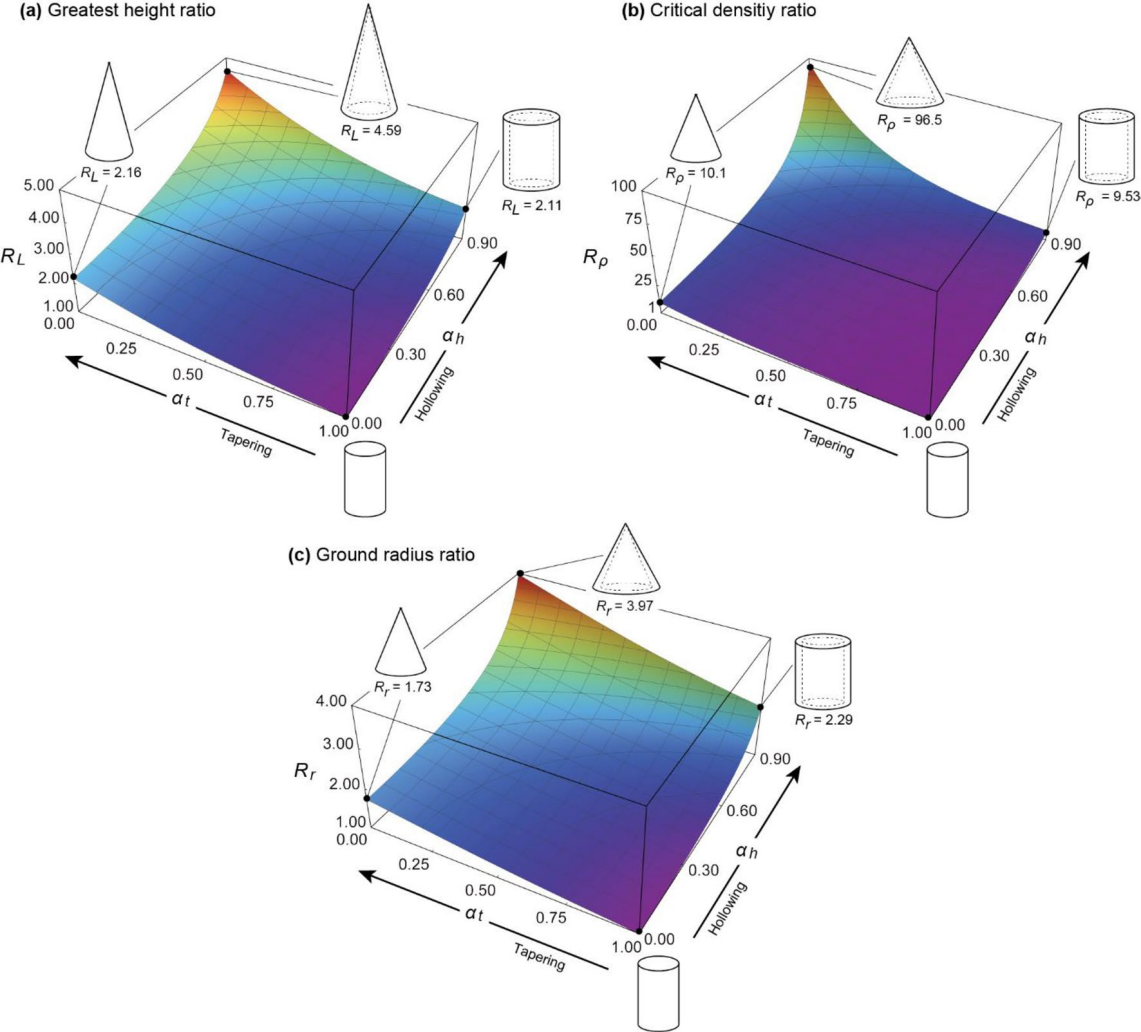
Effect of tapering and hollowing on self-buckling characteristics under constant volume conditions (**a**) Greatest height ratio ${R}_{L}$. It is the ratio of the greatest height in tapering and hollowing models to those of non-tapering and hollowing models. (**b**) Critical density ratio ${R}_{\rho }$. It is the ratio of critical density in tapering and hollowing models to those of non-tapering and hollowing models. (**c**) Ground radius ratio ${R}_{r}$. It is the ratio of ground radius in tapering and hollowing models to those of non-tapering and hollowing models.

Figure 5 shows the effects of tapering and hollowing on the self-buckling characteristics under constant volume conditions. Considering a solid cylinder with radius ${r}_{s}$ and a hollow truncated cone of equal height, and assuming that the volumes of both are equal, the fixed-end radius ${r}_{g}$ of the model with arbitrary taper ratio ${\alpha }_{t}$ and hollow ratio ${\alpha }_{h}$ is given by

29 $$\begin{array}{c}{r}_{g}=\sqrt{\frac{3}{\left(1-{\alpha }_{h}^{2}\right)\left(1+{\alpha }_{t}+{\alpha }_{t}^{2}\right)}}{r}_{s}. \end{array}$$

By substituting Eq. (29) into (25), the critical density ${\rho }_{c}$ can be obtained (Fig. 5b). From this, the ratio of the arbitrarily tapered hollow cone to the critical density of a basic cylindrical column can be calculated and converted to the height ratio to obtain the greatest height ratio ${R}_{L}({\alpha }_{t},{\alpha }_{h})$ in Fig. 5a.

The figure shows that under constant volume conditions, the greatest height can be increased by a maximum of approximately 2.1 times when only hollowing is employed and approximately 2.2 times when only tapering is employed. Furthermore, when both are employed simultaneously, as in the case of bamboos, the greatest height can be significantly increased, reaching approximately 4.6 times that of a solid cylinder. In the case where only one of the two is employed and the volume is constant, a hollow cross-section and a tapered form have the same effect on improving the resistance to self-weight buckling. These trends are similar to those for the critical density, as shown in Fig. 5b.

Moreover, as is evident from Eq. (6), under the constant volume condition, the ground radius ${r}_{g}$ is larger than the radius ${r}_{s}$ of the basic cylindrical column owing to tapering and hollowing. Figure 3 shows this inequality, in which the vertical axis represents the ground radius ratio ${R}_{r}({\alpha }_{t},{\alpha }_{h})$ given by

30 $$\begin{array}{c}{R}_{r}\left({\alpha }_{t},{\alpha }_{h}\right)=\frac{{r}_{g}}{{r}_{s}}=\sqrt{\frac{3}{\left(1-{\alpha }_{h}^{2}\right)\left(1+{\alpha }_{t}+{\alpha }_{t}^{2}\right)}}. \end{array}$$

In the case of distributing the constant volume, the radius at the fixed end ${r}_{g}$ is larger than that of the reference cylinder by a factor of up to 1.7 when only a tapered form is employed and by 2.3 when only a hollow cross-section is employed. When both are employed, the radius is enlarged to approximately 4.0 times that of the reference cylinder. Note that the increase in the base radius has the effect of increasing the moment of resistance to overturning due to lateral loading^13^; however, a larger area must be occupied, and the highest priority should be given to growth in the height direction for the acquisition of light resources.

### Required performance to achieve specific self-weight buckling characteristics

The previous sections considered the types of forms that can obtain high self-weight buckling characteristics under specific conditions. However, trees must simultaneously grow up to a sufficient height to receive light and acquire the self-buckling characteristic that supports their own weight. In this section, we consider the effects of tapering and hollowing on self-buckling characteristics by asking “what properties are required to obtain a specific self-buckling characteristic?”.

**Figure 6:**
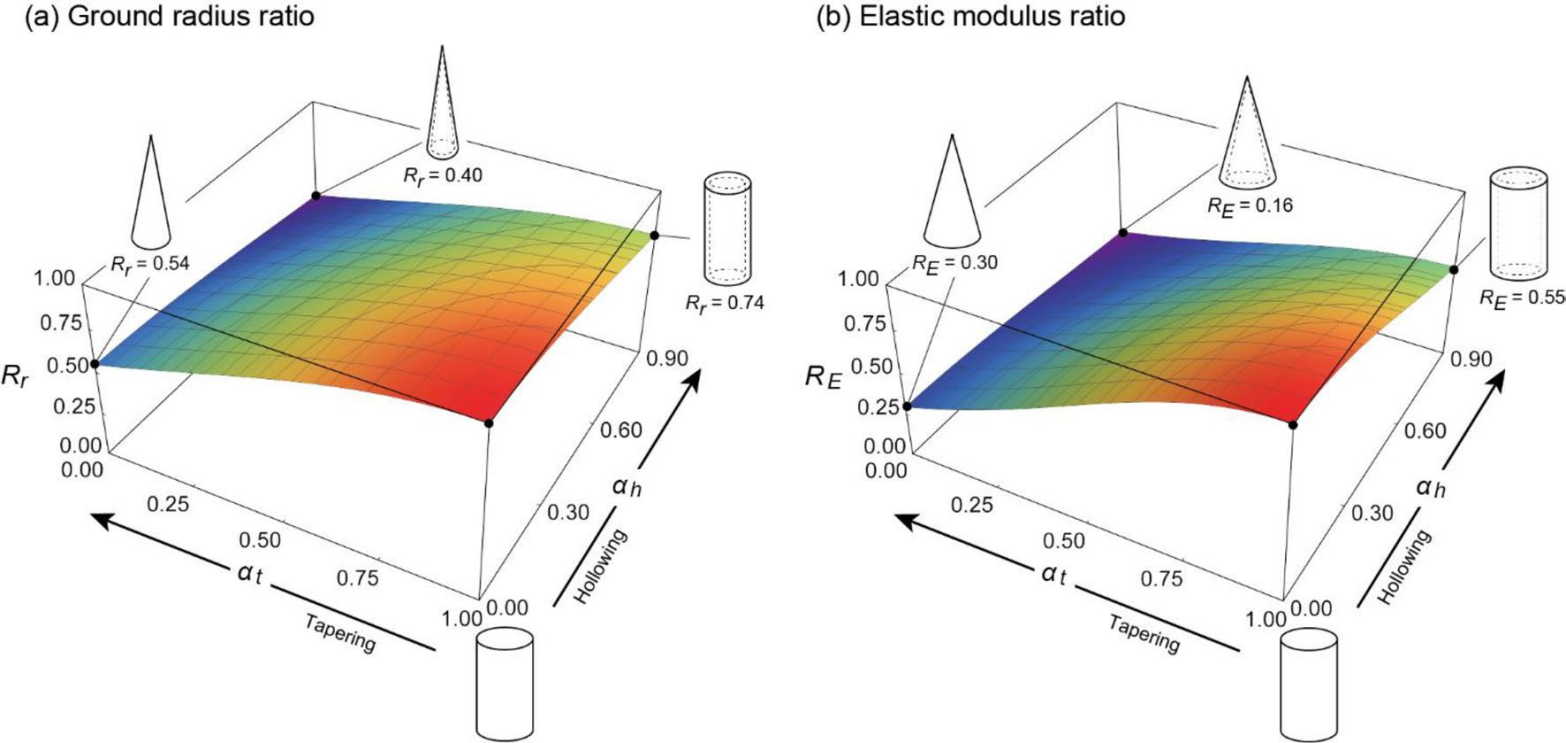
Required properties for constant self-buckling characteristics. (**a**) Ground radius ratio ${R}_{r}$. It is the ratio of ground radius in tapering and hollowing models to those of non-tapering and hollowing models. (**b**) Elastic modulus ratio ${R}_{E}$. It is ratio of ground radius in tapering and hollowing models to those of non-tapering and hollowing models.

Figure 6 shows the required ground radius ${r}_{g}$ and elastic modulus $E$ for realizing the same greatest height as the maximum height in the basic cylindrical column. Therefore, the vertical axes in Fig. 6a,b are respectively obtained as follows:

31 $$\begin{array}{c}{R}_{r}\left({\alpha }_{t},{\alpha }_{h}\right)={\left(f\left({\alpha }_{t}\right)f\left({\alpha }_{h}\right)\right)}^{-3/2}, \end{array}$$

32 $$\begin{array}{c}{R}_{E}\left({\alpha }_{t},{\alpha }_{h}\right)={\left(f\left({\alpha }_{t}\right)f\left({\alpha }_{h}\right)\right)}^{-3}. \end{array}$$

The results for the fixed-end radius ratio ${R}_{r}$, as shown in Fig. 6a, indicate that the same greatest height can be achieved with a fixed-end radius of 0.74 times that of the basic cylinder when only hollowing is employed and 0.54 times that when only tapering is employed. Furthermore, when both are simultaneously considered, the same greatest height can be reached with a fixed-end radius that is 0.4 times that of the basic cylinder. Therefore, tapering and a hollow cross-section can reduce the energy required for radial growth compared with that required by a cylinder, and more energy can be spent on height growth. When either of the two forms is adopted, the tapered form is superior.

Furthermore, a similar trend was observed for the elastic modulus ratio ${R}_{E}$, as shown in Fig. 6b, indicating that to reach the same greatest height, the elastic modulus needs only to be 0.55 times that of the basic cylinder when hollowing is adopted and 0.30 times when tapering is adopted. Furthermore, when both are simultaneously considered, the modulus of elasticity is 0.16 times that of the basic cylinder to reach the same greatest height.

Because the elastic modulus is a mechanical property that is expected to increase with growth owing to lignification and other phenomena, achieving the same height with a smaller elastic modulus is highly advantageous. Considering this, if either tapering or hollowing is employed, tapering can achieve a specific self-weight buckling property at an earlier growth stage.

## Discussion

In this section, we discuss the advantages and disadvantages of tapering and hollowing in plants with respect to their self-weight buckling characteristics. Table 2 lists the key results of this study. It shows that under all the conditions analyzed, using a tapered form is superior to using a hollow cross-section for obtaining self-weight buckling characteristics. Furthermore, using both significantly increases the self-weight buckling characteristics under all conditions. Based on these results, the tapered form should be adopted first when either a hollow cross-section or tapered form is adopted. Although hollow cross-sections increase the growth rate owing to weight reduction, they reduce the resistance to ovalizing and tipping. By contrast, the tapered form is a superior approach that ensures buckling under self-weight conditions without significant mechanical loss. These facts suggest that adopting the tapered form should be the preferred action for any plant that requires height. In fact, observing plant geometry, several representative examples exist for plants that are not hollow, including trees; however, the tapered form is a geometrical feature possessed by most woody and herbaceous plants.

**Table 2:** Summary of calculation results.

Condition			Multiples of values for solid, non-tapering cylinder		
			Only tapering	Only hollowing	Tapering and hollowing
Ground radius and elastic modulus: constant	Greatest height ratio ${R}_{L}$	Density: constant	1.50	1.22	1.82
	Critical density ratio ${R}_{\rho }$	Height: constant	3.38	1.81	6.01
	Critical weight ratio ${R}_{W}$	Height: constant	1.13	0.34	0.39
Volume and elastic modulus: constant	Greatest height ratio ${R}_{L}$	Density: constant	2.16	2.11	4.59
	Critical density ratio ${R}_{\rho }$	Height: constant	10.1	9.53	96.5
	Ground radius ratio ${R}_{r}$	Height: constant	1.73	2.29	3.97
Height and density: constant	Ground radius ratio ${R}_{r}$	Elastic modulus: constant	0.54	0.74	0.40
	Elastic modulus ratio ${R}_{E}$	Ground radius: constant	0.30	0.55	0.16

Moreover, we can assume that survival strategies, such as “plants as individuals” or “grouped plants,” and lifespan are related to the differences in the adoption of hollow and solid cross-sections. Individual plants with long lifespans, such as trees, must be resistant to various forces. Therefore, we assume that because hollowing the cross-section reduces resistance to ovalizing and reduces the moment of fall resistance due to weight reduction, trees only adopted a tapered form. A tapered form lowers the center of gravity and ensures that not only the self-weight and height but also the mechanical properties of the cross-section, such as the moment of inertia, have a gradient. We assume that this allows a gradual increase in the risk of failure toward the free end, thus leading to a reduced risk of “root turnover,” which is fatal to trees.

In contrast to trees, bamboos, which have a shorter lifespan than trees and are connected by underground stems^42^, adopt a strategy of reducing self-weight at the expense of resistance to external forces to increase the number of shoots reaching the ground and ensure rapid growth. Bamboos, a representative example of hollow plants among woody species, simultaneously adopt both a tapered form and hollowing^19^, thus enabling them to reach the same height as trees at an astonishing speed. Furthermore, the tapered form achieves higher self-weight buckling characteristics at a smaller fixed-end radius than hollowing. This is also a major advantage for bamboos growing in clusters because it implies that the ground area occupied by each individual bamboo can be reduced.

## Conclusions

In this study, to clarify the advantages and disadvantages of tapering and hollowing from the perspective of the greatest possible height before self-buckling, we modeled woody plants using tapering or hollow cantilevers, formulated the greatest height before self-buckling, and derived a theoretical formula for the greatest possible height considering tapering and hollowing. Moreover, based on this formula, the findings of this study are as follows:

1. The formula of the greatest height with simultaneous tapered form and hollow cross-section can be expressed as the product of the greatest height formula of a cylinder and functions considering tapered form and cross-section. Therefore, the scaling law of cylinders proposed by Greenhill^1^, which states that the greatest height is proportional to the 2/3 of the power of the ground radius, remains valid even when the tapered form and hollow cross-section are considered. This is consistent with the results of McMahon^2^, thereby affirming the applicability of this law for trees.
2. From the discussions based on the theoretical formula in this study, we concluded that the tapered form allows for a greater height at an earlier growth stage than that for hollow cross-section. Therefore, because all plants must receive sufficient light to grow as first, this formula theoretically explains why almost all plants exhibit a tapered form.
3. The hollow cross-section is another effective way for securing same self-buckling characteristics as of the tapered form. However, it reduces the resistance to ovalizing and tipping. This suggests that the survival strategies, such as “plants as individuals” or “grouped plants,” and lifespan are related to the differences in the adoption of hollow and solid cross-sections.

The present results can be expected to contribute to not only design and structural calculations for civil-engineering structures but also forest management and biomass estimation. However, because local buckling is avoided by internodes in a bamboo, the appearance of local buckling with lower thickness were ignored, and we focused only on the self-buckling problem in a beam in this study. In addition, the existence of tip compressive force in general buckling problems were not considered in this study. Moreover, the validation of the greatest-height formula was not performed using real plants; only estimation was performed based on the result of previous research. In future work, we will perform the formulation based on the elastic theory and numerical analysis based on the finite element method considering the local buckling problem, comparing local buckling characteristics with self-buckling characteristics from the viewpoint of the greatest height, and the present contributions will be enhanced for more applied and practical studies.

## Data Availability

The datasets generated and/or analyzed during the current study are available from the corresponding author upon reasonable request.
